# Microglial internalization and degradation of pathological tau is enhanced by an anti-tau monoclonal antibody

**DOI:** 10.1038/srep11161

**Published:** 2015-06-09

**Authors:** Wenjie Luo, Wencheng Liu, Xiaoyan Hu, Mary Hanna, April Caravaca, Steven M. Paul

**Affiliations:** 1 Appel Alzheimer’s Disease Research Institute, Feil Family Brain and Mind Research Institute, Weill Cornell Medical College of Cornell University, 413 East 69^th^ Street, New York, NY10021.

## Abstract

Microglia have been shown to contribute to the clearance of brain amyloid β peptides (Aβ), the major component of amyloid plaques, in Alzheimer’s disease (AD). However, it is not known whether microglia play a similar role in the clearance of tau, the major component of neurofibrillary tangles (NFTs). We now report that murine microglia rapidly internalize and degrade hyperphosphorylated pathological tau isolated from AD brain tissue in a time-dependent manner *in vitro*. We further demonstrate that microglia readily degrade human tau species released from AD brain sections and eliminate NFTs from brain sections of P301S tauopathy mice. The anti-tau monoclonal antibody MC1 enhances microglia-mediated tau degradation in an Fc-dependent manner. Our data identify a potential role for microglia in the degradation and clearance of pathological tau species in brain and provide a mechanism explaining the potential therapeutic actions of passively administered anti-tau monoclonal antibodies.

Alzheimer’s disease (AD), the leading cause of dementia among the elderly, has an associated pathognomonic neuropathology, which includes amyloid plaques and neurofibrillary tangles (NFTs) as well as a concomitant loss of neurons and synapses. Recent large genome-wide association studies (GWAS) have both confirmed and extended a large list of genetic risk factors for late-onset Alzheimer’s disease (LOAD)^1^. While most of these genetic variants contribute only modestly to the overall risk of developing LOAD, many of these genes encode proteins that are known to modulate innate immunity and specifically the function of microglia^2^. Accumulating evidences suggest that microglia and the genetically-determined phagocytic and degradative functions of microglia are one of the major determinants of brain Aβ clearance and amyloid burden, thus contributing to the pathogenesis of LOAD^3^,^4^,^5^,^6^,^7^.

Although tau is primarily an intracellular protein normally bound to microtubules and critical for promoting microtubule assembly and stability, more recent work has confirmed the presence of substantial soluble extracellular tau in brain interstitial fluid as well as the release of tau from neurons following depolarization^8^,^9^. Microglia have been shown to co-localize with both amyloid plaques and NFTs although their exact role in plaque and (or) tangle formation is unclear^10^,^11^,^12^,^13^. Misfolded aggregated forms of extracellular tau have also been shown to be taken up by neurons via endocytosis and to act as seeds that readily induce misfolding and aggregation of intracellular soluble tau and the subsequent intercellular spread of tau aggregates both *in vitro* and *in vivo*^14^,^15^,^16^,^17^,^18^,^19^,^20^,^21^. Whether or not microglia play a role in tau clearance and spread in brain has to our knowledge never been studied.

In mouse models of tau-dependent neurodegeneration, passive immunization with certain anti-tau monoclonal antibodies has also been shown by several laboratories to reduce age-dependent tau pathology, including NFTs, neurodegeneration and behavioral impairment^22^,^23^,^24^,^25^,^26^,^27^,^28^,^29^. How passive immunization with anti-tau monoclonal antibodies reduces age-dependent tau pathology in these mouse tauopathy models is however poorly understood.

We now report that primary mouse microglia rapidly and efficiently internalize and degrade hyperphosphorylated pathological tau isolated from AD brain tissue. We also show that co-incubation of microglia with an anti-tau monoclonal antibody previously shown to effectively reduce the development of NFTs in a human tauopathy mouse model following passive immunization^29^,^30^, markedly enhances microglia-mediated uptake and degradation of pathological tau, a process that requires Fc effector function.

## Results

### Microglia rapidly internalize and efficiently degrade sarkosyl-insoluble tau in a time-dependent manner

**Figure 1:**
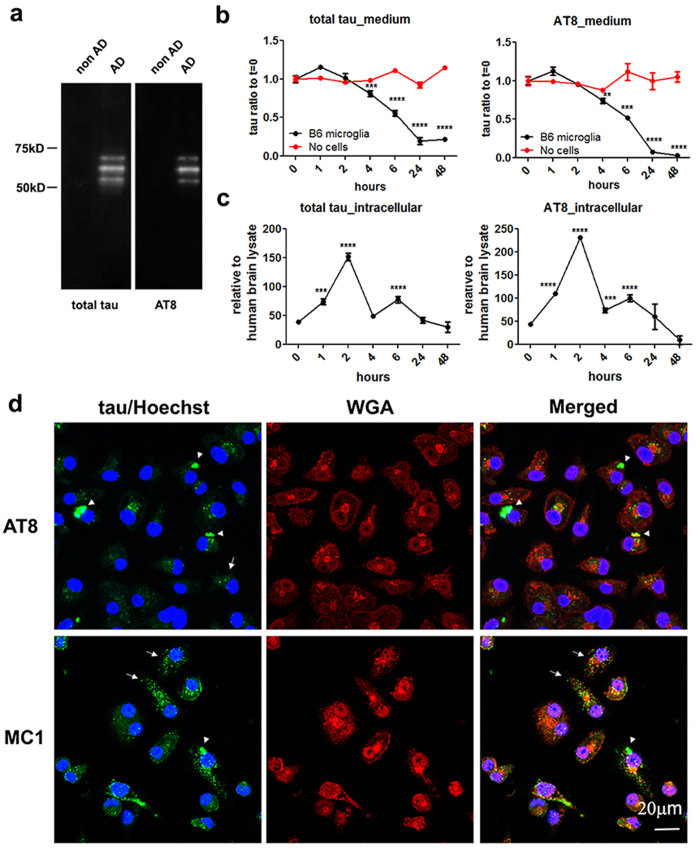
Sarkosyl insoluble tau (SI-tau) is internalized and degraded by microglia. (**a**) The SI fraction isolated from postmortem AD brain, but not from non-AD (control) brain, is enriched in hyperphosphorylated PHF-tau species as shown as three major bands by western blots using tau10 and AT8 antibodies. (**b** and **c**) Primary microglia cultured from postnatal day 1–3 wild-type C57BL/6 mice were incubated with 1 μg/ml SI-tau for the indicated times. The levels of total tau and AT8 positive-tau in the media (**b**) and cells (**c**) were determined by tau specific ELISAs. Cells were subjected to 5 min trypsin digestion to deplete surface bound SI-tau before lysis. Statistical analyses were performed by student *t-test* (unpaired two tails) by comparing samples incubated with microglia to that incubated with media alone in (**b**) and by comparing different time points to 0 h time point (mean ± s.e.m.) (**c**). **p < 0.01 ***p < 0.005 ****p < 0.001. Note the dramatic reduction in total and p-tau in the media following incubation with microglia compared to medium alone (**b**). Note the early increase in internalized tau and subsequent decrease over time (**c**). (**d**) Internalization of SI-tau by microglia is shown by confocal microscopy using indirect immunofluorescence. Primary murine microglia were incubated with 1 μg/ml SI-tau for 120 min and imaged using confocal microscopy after being immunostained with AT8 or MC1 antibody. Plasma membrane was stained by red-fluorescent Alexa Fluor594 wheat germ agglutinin (WGA) and nuclei were stained by blue Hoechst 33342 dye. Note the intracellular localization of AT8 or MC1-positive tau puncta (arrows) in microglia. Large tau aggregates attached to microglial surface were also observed (arrowheads). Scale bar represents 20 μm.

To investigate if microglia play a role in tau degradation and clearance, we first isolated the sarkosyl-insoluble fraction (SI fraction) from postmortem AD brain tissue highly enriched in paired helical filament (PHF)-tau that is hyperphosphorylated and well regarded as pathogenic^31^. We termed this tau species sarkosyl-insoluble tau (SI-tau) in the current study (Fig. 1a). We next incubated SI-tau with primary postnatal mouse microglia isolated from wild-type C57BL/6 mice (≥98.7% purity as determined by immunostaining using FITC-labeled CD11b antibody) for indicated times. As shown in Fig. 1b, both extracellular tau species dramatically decreased in a time-dependent manner after incubation with microglia, with ≤20% total tau and ≤5% phosphorylated tau at Ser202/Thr205 (detected by AT8, a monoclonal antibody recognizing AD-related phosphorylation at Ser202/Thr205 on tau protein) remaining in the media (p < 0.001) after incubation for 48 hrs (Fig. 1b). Incubation of SI-tau with the medium alone in the absence of microglia over the same time period did not result in any significant decrease of total tau or AT8-positive tau (Fig. 1b). Concomitantly, we detected a corresponding and rapid increase of microglia-associated tau (total tau and AT8-positive tau) within 1–2 hrs of incubation followed by a decrease with longer incubation time, suggesting a time-dependent uptake and degradation of tau by microglia (p < 0.001) (Fig. 1c). Using monoclonal antibodies recognizing different phosphorylated tau epitopes, AT8 or MC1 (specific for conformational epitopes on PHF tau), we observed tau inside of microglia as intracellular puncta (arrows) using confocal microscopy after 120 min of incubation (Fig. 1d). These internalized tau puncta were also positively stained by two other antibodies specific for phosphorylated tau, AT180 (recognizing phosphorylated tau at Thr231) and AT270 (recognizing phosphorylated tau at Thr181) (supplementary Figure S1). We also noticed that some large tau aggregates (with sizes varying from 1 μm to 10 μm) are attached to the surface of microglia (arrowheads) (Fig. 1d and supplementary Figure S1). These data demonstrate that microglia readily internalize and degrade hyperphosphorylated tau from human AD brain.

### Microglia degrade hyperphosphorylated tau and NFTs *ex vivo*

**Figure 2:**
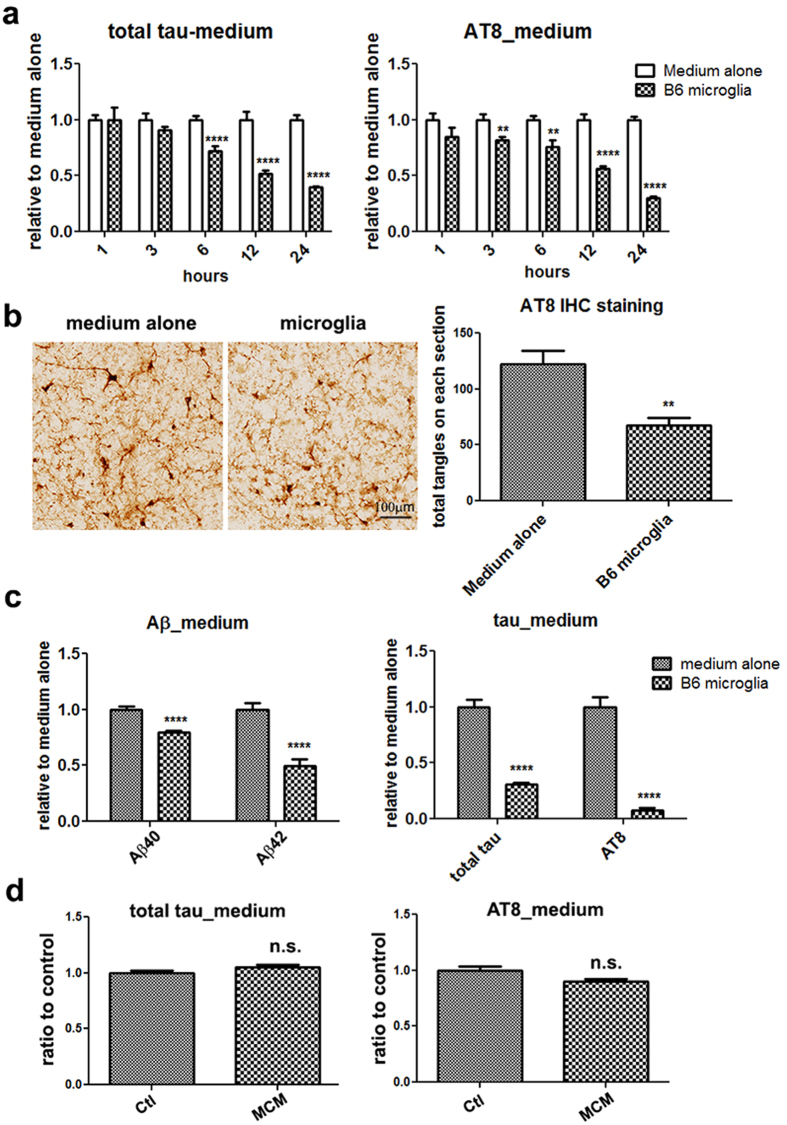
Microglia efficiently degrade brain-derived phosphorylated tau and neurofibrillary tangles *ex vivo*. (**a** and **b**) Frozen brain sections (10 μm) from 6-7 month old tau P301S homozygous transgenic mice were incubated with or without C57BL/6 mouse microglia for the indicated times. The level of total tau and AT8 positive-tau in the medium decreased in a time-dependent manner in the presence of microglia compared to medium alone control (**a**). Representative P301S transgenic mouse brain sections incubated with microglia or medium alone for 24 hrs. NFTs in each brain section were visualized by IHC with the AT8 antibody (NFTs in thalamus, left) and the total NFTs in hippocampal, frontal cortical and brain stem (thalamus) were quantified using Nikon Elements software program (right). *n* = 8 sections/treatment group (**b**). Note the significant decrease in NFTs (*p ≤ 0.01*) following incubation with primary microglia (**b**). (**c**) Frozen tissue sections from an advanced AD brain (Braak stage IV) were incubated with microglia for 24 hrs. The levels of Aβ40 and Aβ42 (left), total human tau and AT8 positive-tau (right) in the medium were determined by ELISAs. Note the marked decrease of Aβ42, total tau and AT8-positive tau in the medium following incubation with primary microglia. Statistical analyses were performed by student *t-test* (unpaired two-tails) (mean ± s.e.m.). **p < 0.01; ***p < 0.005; ****p < 0.001. Scale bar represents 100 μm. (**d**) Conditioned medium of microglia has no tau-degrading activity. Frozen brain sections from a 6-7 month old P301S transgenic mouse were incubated with control medium (Ctl) or microglia-conditioned medium (MCM) for 24 hrs. Total human tau (left) and AT8-positive-tau (right) in the medium were measured by ELISAs. Incubation with MCM had no effect on tau degradation.

To examine whether microglia can degrade hyperphosphorylated tau species that are derived directly from brain tissues with NFTs, mouse microglia were added on top of unfixed frozen brain sections (10 μm thickness) prepared from 6–7 month P301S transgenic mice, containing abundant NFTs that are predominantly composed of hyperphosphorylated tau as described previously^32^. Following incubation with medium at 37 °C, hyperphosphorylated tau proteins (represented by AT8-positive tau) are passively released from these thin tissue sections (free tau species); the medium concentration of tau (total tau and AT8-positive tau) quickly reached a plateau after 1 h and levels were maintained through 48 hrs (supplementary Figure S2a). When brain sections were incubated with microglia for 1 hr, no significant difference in the medium levels of free tau proteins was observed compared to the medium alone group (Fig. 2a and supplementary Figure S2b). With longer incubation time, however, we observed a time-dependent decrease in the amount of tau in the medium but only in the presence of microglia, i.e. compared to medium alone (Fig. 2a). After 24 hrs of incubation, ≤40% total human tau and ≤30% AT8 positive-tau relative to medium alone were detected in the medium containing microglia (p < 0.001) (Fig. 2a). These data demonstrate that mouse microglia can efficiently degrade free tau species released into the medium from P301S mouse brain sections. Using quantitative immunohistochemistry (IHC), we also observed a significant reduction in the number of NFTs present in brain sections after incubation with microglia for 24 hrs compared to brain sections incubated with medium alone (p < 0.01, *n* = 8, Fig. 2b). Thus, microglia also efficiently eliminate NFTs present in P301S mouse brain sections.

Next, we examined the tau-degrading activity of microglia employing brain sections (10 μm) prepared from unfixed frozen frontal cortex of AD patients at an advanced stage of their disease (Braak stage IV). Consistent with the previous observation of Aβ degradation by microglia^6^, the levels of Aβ40 and Aβ42 present in the medium were significantly reduced in the presence of microglia compared to medium alone after 24 hrs (*p ≤ 0.001*, Fig. 2c, left). Strikingly, we also observed a dramatic reduction of total tau (≥70%) and AT8 positive-tau (≥90%) in the medium after 24 hrs of incubation with microglia compared to the medium alone (*p ≤ 0.001*, Fig. 2c, right). Due to the postmortem interval and storage time for the AD brain tissue samples, we could not obtain good histological images for quantification of the number of amyloid plaques or NFTs remaining in the AD brain sections. Nonetheless, these data demonstrate that microglia can very efficiently degrade not only Aβ peptides but also phosphorylated tau species released from AD brain section.

We have previously shown that enzymes secreted by macrophages, which share the same lineage with microglia, can degrade both Aβ and amyloid and contribute to amyloid clearance^4^. To address whether proteases secreted by microglia also play a role in microglia-induced tau degradation, we collected conditioned media that had been cultured with microglia for 24 hrs (MCM) and then incubated it with P301S transgenic mouse brain sections for another 24 hrs. No significant decrease in the levels of either brain-released total tau or AT8-positive tau was observed in MCM comparing to normal medium following 24 hrs incubation (*p = n.s.;* Fig. 2d). These results suggest that enzymes secreted from microglia contribute little if any to microglial-induced tau degradation and that the presence of microglia (and likely the internalization of tau) is necessary for tau degradation.

### FITC-AT8-tau complex is readily internalized by live microglia

**Figure 3:**
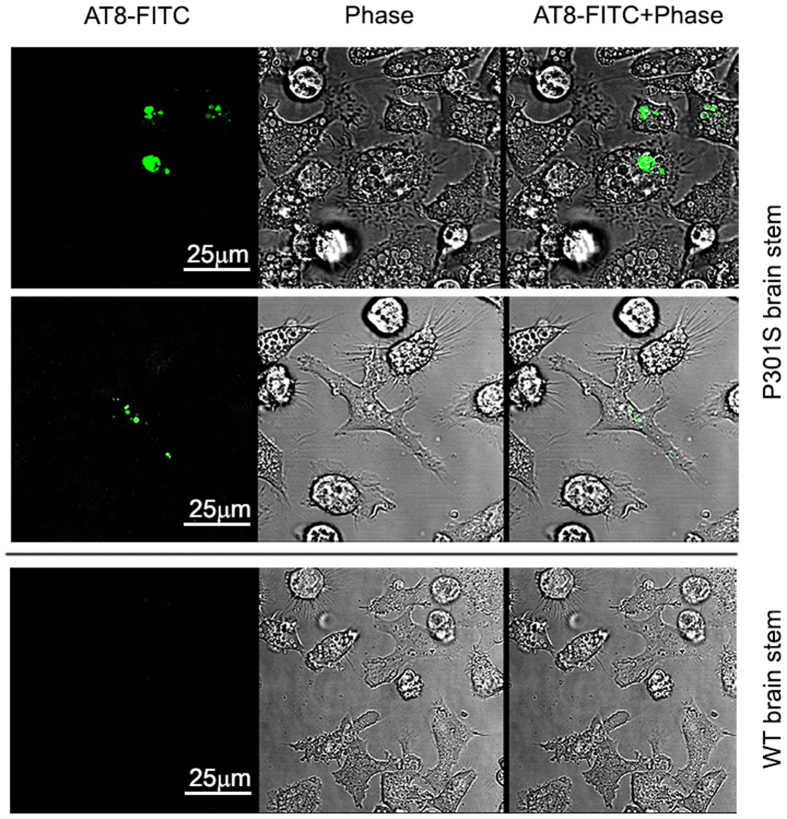
Uptake of the FITC-AT8-tau complex by live microglia. Fresh brain stem sections from P301S transgenic mice or age-matched wild type C57BL/6 mice were labeled with FITC-conjugated AT8 antibody as described in the Methods. After removing extra FITC-AT8, the labeled tissue was then co-incubated with microglia for 60 minutes. Live images of microglia were collected near brain section area using confocal microscope. No signals were detected in microglia incubated with brain stem tissues from wild type mouse. Scale bar represents 25 μm. Note the marked internalization of the FITC-AT8-tau complex by microglia.

Passive immunization with certain anti-tau monoclonal antibodies has been shown to reduce age-dependent tau pathologies in several tauopathy mouse models^22^,^23^,^24^,^25^,^26^,^27^,^28^,^29^. We next examined the ability of live microglia to internalize pathologic tau derived from P301S mouse brain in the presence of anti-tau monoclonal antibody AT8 conjugated with FITC. Fresh brain stem tissue sections were prepared from P301S transgenic mice and incubated with the FITC-conjugated AT8 antibody in the presence of 1% Triton x-100. After extensive washing in phosphate buffer to remove unbound FITC-labeled antibody, we then placed the tissue sections with FITC-AT8-bound tau into the chamber seeded with microglia. After co-incubation for 60 minutes, the FITC-tau signal was visualized in large organelles inside of microglia located near the brain sections using live cell confocal imaging (Fig. 3). By contrast, no FITC was detected in microglia after incubation with brain stem tissue sections prepared from wild type mice and subjected to FITC-AT8 incubation (Fig. 3). These data demonstrate that the FITC-AT8-tau complex is readily internalized by live microglia, indicating that anti-tau antibodies may enhance tau internalization and degradation by microglia.

### The anti-tau monoclonal antibody MC1 facilitates tau degradation by microglia in an Fc-dependent manner

**Figure 4:**
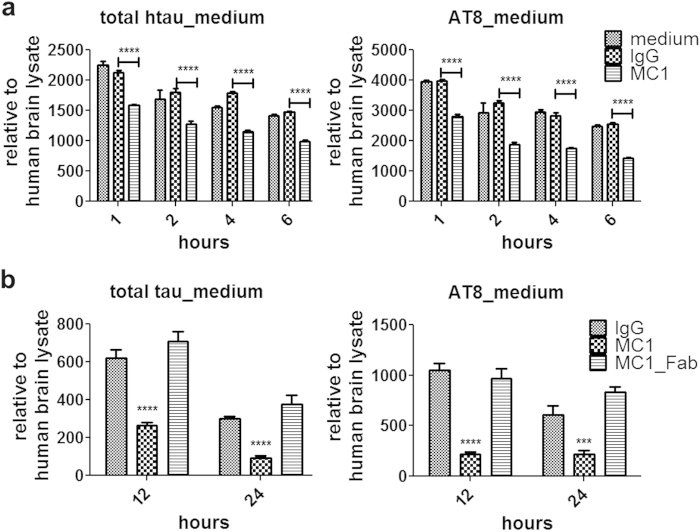
Tau degradation by microglia is facilitated by the anti-tau monoclonal antibody MC1 in an Fc-dependent manner. (**a**) Microglia were incubated with 1 μg/ml SI-tau for 1, 2, 4, and 6 hrs in the presence or absence of 2.5 μg/ml IgG1 or MC1 antibody; MC1 but not a non-immunized IgG1 enhanced microglia-mediated tau degradation at all time points examined. (**b**) The MC1 Fab was devoid of activity in promoting microglia-mediated tau degradation. Microglia were incubated with 1 μg/ml SI-tau for 12 or 24 hrs in the presence of 2.5 μg/ml IgG, MC1 or MC1 Fab. The levels of total tau or AT8 positive-tau were determined by ELISAs. Statistical analysis was performed by student *t-test* (unpaired two-tails) (mean ± s.e.m.). ***p < 0.005; ****p < 0.001.

To better understand how anti-tau antibodies might reduce tau pathology following passive immunization, we next examined if anti-tau antibodies can facilitate microglia-mediated tau degradation. We used MC1, a conformation-dependent tau antibody that recognizes an early pathological tau conformation^29^,^33^ and that has been previously shown by several groups to reduce age-dependent tau pathology when passively administrated to tau transgenic mice^29^,^30^. After primary microglia were incubated with SI-tau in the presence or absence of MC1 or a control mouse IgG (non-immunized), a significant enhancement of microglial-dependent tau degradation (for both total tau and AT8 positive-tau) was observed in a time-dependent manner in the presence of MC1 compared to the IgG control antibody (Fig. 4a). These data demonstrated that MC1 promotes tau degradation mediated by microglia. We next examined whether Fc effector function is necessary for MC1-mediated tau degradation by microglia. As shown in Fig. 4b, SI-tau degradation by microglia was greatly enhanced by MC1 after incubation for 12 and 24 hrs. By contrast, the MC1 Fab (without the Fc domain) was unable to enhance tau degradation by microglia. These results suggest that Fc effector function is necessary for the antibody-enhanced internalization and degradation of tau by microglia.

## Discussion

In our study we demonstrate that murine microglia can efficiently internalize and degrade pathological tau species derived from human AD and P301S transgenic mouse brain. Our data thus suggest that microglia may play an important role in the clearance of both native and pathological tau species in the central nervous system. Further, the ability of microglia to internalize and degrade tau is enhanced by the anti-tau monoclonal antibody MC1 via an Fc-dependent mechanism, revealing a potential mechanism for antibody-based tau immunotherapy.

Although microglia have been repeatedly implicated in the pathogenesis of various neurodegenerative disorders, including tauopathies such as FTD and AD^34^, their exact role is still a matter of debate. Among a number of plausible mechanisms, reduced Aβ phagocytosis and clearance represents a distinct possibility as recently proposed and further supported by numerous GWAS data^34^. Among these newly identified AD-risk genes, many are in fact essential regulators of innate immunity, including the antigen-presenting and phagocytic and degradative functions of circulating macrophages and microglia^3^,^6^,^35^,^36^,^37^,^38^. These findings suggest that genetically-determined and (or) age-related impaired microglial phagocytosis may more generally underlie the pathogenesis of a number of neurodegenerative disorders including AD and FTD.

Based on our findings, we propose that microglia play a role in the clearance of extracellular tau in brain and impaired microglia-mediated phagocytosis and degradation of tau may contribute to tau pathology in AD and FTD. In fact, earlier studies have observed that the regional distribution of cortical microglia parallels that of NFTs in AD brain^39^ and microglia have also been shown to infiltrate extracellular NFTs^40^. Moreover, activated microglia have been observed to be more significantly correlated with the development of NFTs than with amyloid-containing senile plaques^10^. While it still remains a matter of controversy how microglia affect tau pathology in AD, some studies suggest that microglial activation by proinflammatory cytokines/chemokines in AD brain appears to induce tau phosphorylation thereby promoting tau aggregation and NFT formation^41^. However, Streit and coworkers have proposed an alternative model that age-related microglial degeneration or senescence and thus a loss of the neuroprotective properties of microglia contribute to AD pathogenesis and not microglial activation per se^42^. On the basis of their histopathological analysis, these investigators have reported that dystrophic microglia display cytoplasmic abnormalities, such as de-ramified, fragmented, and bulbous swelling, are colocalized with NFTs and neuropil threads characteristic of degenerating neurons and that the presence of these dystrophic microglia precedes NFT formation and neurodegeneration in AD brain^42^. These findings are particularly noteworthy in light of our data demonstrating that microglia (prepared from young mice) very efficiently internalize and degrade tau. In healthy human brain, microglia serve several important roles and notably respond to pathological insults via their effector functions including phagocytosis and degradation^43^. During aging, microglia generally show diminished phagocytosis as well as a hyperreactive proinflammatory phenotype^43^. The status of microglia in late AD brain may therefore represent an exaggerated state of aging or senescent microglia^43^. Our *in vitro* and *ex vivo* data showing that neonatal microglia can very efficiently internalize and degrade pathologic tau support the model that the impaired phagocytic function of microglia in AD may result in the brain will cause accumulation and inter-neuronal spread of pathological tau species, thus contributing to the progression of tau pathology and neurodegeneration in AD. Further studies using both *in vitro* and especially *in vivo* models will be necessary to validate this hypothesis. A comparison of tau degrading activity of young vs. old microglia as well as from microglia isolated from normal and pathologic AD brain could be particularly informative.

The ability of microglia to degrade Aβ is well established^6^. Microglia are also capable of clearing Aβ through the production and in some cases the release of various Aβ degrading enzymes, such as insulin-degrading enzyme (IDE), neprilysin (NEP), and matrix metalloprotease 9 (MMP9), which are secreted to degrade Aβ extracellularly^44^. By contrast, we found that the conditioned media of microglia contains little tau degrading activity. This result suggests that the microglia-mediated tau degradation is largely carried out following internalization most likely via a phagocytic mechanism and that extracellular clearance by secreted microglial enzymes contributes very little to tau degradation. Whether Aβ and tau degradation by microglia share the same cellular mechanism(s) requires further investigation.

Many of the AD-risk genes identified by GWAS encode proteins that are expressed by microglia and have been shown to regulate innate immunity, including the antigen-presenting and phagocytic as well as degradative functions of circulating macrophages and microglia^3^,^35^,^36^,^37^,^38^. Genetic variants of genes such as CD33 and TREM2 have been shown to alter (increase or decrease) the phagocytic functions of microglia and thus may directly (or indirectly) contribute to tau pathology, independent of their potential effects on amyloid pathology. Our results suggest that these genetic variants may impact AD by simultaneously and possibly independently impacting both tau and amyloid pathology.

Encouraging results using passively administered anti-tau monoclonal antibodies to reduce tau pathology in mutant tau transgenic mice have been reported^22^,^23^,^24^,^25^,^26^,^27^,^28^,^29^. How passive immunization with anti-tau antibodies reduces tau pathology is however poorly understood. One possibility is that these antibodies enhance the clearance of extracellular tau species, reducing the intercellular spread and transmission of tau aggregates as tau pathology progresses neuro-anatomically over time. The facilitation of tau phagocytosis and degradation by microglia may therefore be one mechanism by which these antibodies reduce tau pathology. Here we show that one such antibody MC1, previously shown to reduce tau pathology following passive immunization of P301L and P301S mice^29^,^30^ markedly enhances tau degradation by microglia *in vitro*. Moreover, the increased tau degradation by microglia induced by MC1 appears to require effector function as the MC1 Fab is inactive, again implicating microglia in the mechanism of action of MC1 and potentially other anti-tau antibodies. This finding is reminiscent of earlier studies showing that administration of monoclonal or polyclonal antibodies directed against Aβ profoundly alters microglial phenotype and can induce Aβ/amyloid phagocytosis and degradation by microglia via the Fc domain dependent manner^45^,^46^.

Our data raise the interesting possibility that both pathognomonic lesions of AD, Aβ/amyloid-containing plaques and NFTs may independently result from impaired microglial function, specifically the ability of microglia to phagocytize and degrade or clear extracellular Aβ and tau species that are prone to aggregate and form either extracellular amyloid plaques or intracellular NFTs. We speculate that genetic and age-related factors that impair the phagocytic function of microglia could increase the risk of developing AD and perhaps other tauopathies. Finally, our studies suggest that stimulating the phagocytic and degradative function of microglia using small molecules or antibodies may represent a useful therapeutic strategy for a number of neurodegenerative diseases, i.e. to facilitate the microglia-mediated degradation and clearance of proteins that are prone to misfold or aggregate.

## Experimental Procedures

### Ethics Statement

All experiments were conducted in accordance with relevant NIH guidelines and regulations, related to the Care and Use of Laboratory Animals and human tissue. Animal procedures were performed according to protocols approved by the Research Animal Resource Center at Weill Cornell College of Medicine.

### Animals

Homozygous P301S tau transgenic mice (kindly provided by Dr. Michel Goedert, Cambridge, United Kingdom) expressing the P301S human 0N4R isoform (383aa) under Thy1.2 promoter were maintained and characterized previously^32^. These mice were intercrossed and maintained on a C57BL/6J background.

### Human material

Frontal cortical tissue samples from AD individuals or non-AD age-matched controls were obtained from the Banner Sun Health Research Institute (Sun City, AZ), Kathelene Price Bryan Brain Bank, Duke University, and Dr. Bernardino Ghetti of Indiana University.

### Isolation of the sarkosyl-insoluble fraction from human brain

The sarkosyl-insoluble fraction of AD or non-AD frontal cortical tissue was prepared using the method described by Greenberg and Davies with minor modification^31^. Briefly, brain tissue was homogenized with cold homogenization buffer (10 mM Tris/1 mM EGTA/0.8 M NaCl/10% sucrose, pH7.4) in a Teflon glass homogenizer. The resulting brain homogenate was then centrifuged at 27,200 × g for 20 min at 4 °C. The resulting supernatant was subjected to extraction with 1% (wt/vol) N-lauroylsarcosine in the presence of 1% (vol/vol) 2-mercaptoethanol at 37 °C for 2–2.5 hrs followed by centrifugation at 108,000 × g for 30 min at room temperature. The pellet recovered from the centrifugation was quickly rinsed three times with PBS and then dissolved in PBS and stored at −80 °C. This preparation is called the sarkosyl-insoluble fraction (SI-tau). The amount of total tau protein in the sarkosyl insoluble fraction was quantified by western blot using HT7 antibody (Thermo-Scientific) using a standard curve generated with recombinant tau protein purified from E.coli (Sigma).

### Human tau ELISA

Human total tau and pS202/T205-tau were measured by specific sandwich ELISAs as described in Chai *et al.*^29^. Briefly, 96-well plates were pre-coated with 5 μg/ml AT8 (Thermo Scientific) or Tau10 (a gift from Tom Malia, Johnson and Johnson) overnight at 4 °C followed by blocking with StartingBlock blocking buffer (Thermo Scientific/Pierce). Samples of medium or cell lysate were diluted in Superblock blocking buffer (Thermo Scientific/Pierce) and loaded onto the plates together with biotinylated-HT7 antibody (Thermo Scientific #MN1000B, 1:200). After incubation for 2 hrs at room temperature, samples were then washed five times with TBS/0.5% Tween 20 wash buffer followed by incubation with streptavidin-HRP (Jackson Immunoresearch #016-030-084) for 30 min. Following this, the plates were developed by incubating with one-step TMB substrate (Thermo Scientific) for 30 min and stopped by 2N H_2_SO_4_ and then read using BioTek Synergy H1 hybrid Reader at 450 nm. The quantity of AT8- or Tau 10-reactive tau was determined using a standard curve derived from human AD brain homogenates, and plotted as a relative amount of AD brain homogenate.

### Human Aβ ELISAs

Aβ levels in the media of AD brain sections after incubation with or without microglia were assayed as previously described^4^. In brief, the culture medium was diluted by two-fold with Startingblock blocking buffer (Thermo Scientific/Pierce) and then loaded onto plates pre-coated with an antibody that specifically recognizes the C-terminal domain of Aβ42 (21F12) or Aβ40 (2G3) (kindly provided by Dr. Ronald Demattos, Eli Lilly). After incubation for 2 hrs, samples were washed four times with TBS washing buffer containing 0.5% Tween20 and then incubated with biotinylated 3D6 antibody as a detecting antibody (recognizing the N-terminus of Aβ42 and Aβ40) for one hour. The remainder of the ELISA followed the same protocol as described above for the human tau ELISA. Aβ42 and Aβ40 were determined using a standard curve derived from synthetic Aβ42 or Aβ40 peptides (Ana-Spec). All procedures were carried out at room temperature.

### Isolation and culture of primary microglia

Primary postnatal microglia were prepared as previously described^47^. Cerebral cortices from 1–3 day old neonatal C57BL/6 mice were dissected, stripped of their meninges and digested for 30 min with 0.25% trypsin at 37 °C. Trypsinization was stopped by the addition of an equal volume of culture medium (Dulbecco’s modified Eagle medium: F-12 nutrient mixture: fetal bovine serum 45:45:10, penicillin 100 U/mL and streptomycin 100 μg/mL containing 0.02% deoxyribonuclease I). The cell suspension was pelleted, resuspended in culture medium and finally suspended into a single-cell suspension by repeated pipetting followed by passage through a 40 μm pore mesh. Cells were seeded into a 75 ml flask and cultured in culture medium containing 50 ng/ml GM-CSF at 37 °C in humidified 5% CO_2_−95% air. Cells were harvested at 12 DIV by shaking for 2 hrs at 200 rpm. Floating microglia were pelleted and seeded onto 48 well (3 × 10^5^/well) or 24 well (6 × 10^5^/well) culture plates in culture medium for each experiment. Serum-free medium containing 0.2%BSA was used for the tau degradation experiments.

### Purification of MC1 Fab

The MC1 Fab was prepared using a Pierce^TM^ Fab Micro preparation kit following the manufacture’s protocol (Thermo-Scientific). Briefly, the MC1 IgG was digested with agarose-immobilized papain at 37 °C for 5 hrs to generate a mixture of 50 kD Fab and Fc fragments. After centrifugation, protein A resin was added to the supernatant to capture the Fc molecules. The unbound Fab was then recovered from the supernatant after removing the protein A resin by centrifugation at 1000 × g. The supernatant containing the Fab was further concentrated using a concentrator (Amicon). The concentrations of MC1 IgG and MC1 Fab were determined by OD280 and confirmed by silver staining after SDS-PAGE.

### *In vitro* SI-tau degradation assay

Primary microglia were plated in 48-well plates in culture medium at a density of 3 × 10^5^ cells/well. The cells were cultured overnight and then switched to serum-free media/0.2% BSA containing 1 μg/ml SI-tau and incubated for the indicated treatment times. After incubation, the culture medium was collected, total tau and p202/T205-tau levels were measured by the tau specific ELISA as described above. The cells were washed once with PBS and then trypsinized with 0.25% trypsin for 5 min before being lysed into 100 μl of RIPA buffer (Sigma). The levels of intracellular tau species in RIPA lysed cells were measured by ELISA after two-fold dilution with Superblock buffer (Thermo Scientific/Pierce). To test the activities of MC1 or MC1 Fab on microglia-mediated tau degradation, microglia were incubated with 1μg/ml SI-tau for the indicated times in the presence of 2.5 μg/ml mouse IgG1, or the anti-tau antibody MC1 (provided by Dr. Peter Davies) or purified MC1 Fab. After incubation, tau species remaining in the media were measured by ELISA as described above.

### *Ex vivo* assays

Brains of homozygous transgenic P301S mice at 6-7 months of age were quickly removed and frozen in dry ice. Coronal sections (10 μm) were continuously cut on a MICROM HM550 cryostat (Thermo Scientific), mounted onto poly-l-lysine coated 12 mm round coverslips (BD BioCoat #354085), transferred to a 24-well culture plate in an arrangement such that tissue sections used for the medium alone and microglia incubation are anatomically adjacent to each other. Brain sections were used immediately or stored at −80 °C until use. Freshly harvested primary microglia at 12DIV were seeded on top of brain sections at a density of 6 × 10^5^ cells/well in 0.5 ml of microglia culture medium and incubated at 37 °C for the indicated times. For the *ex vivo* assay with AD brain sections, 10 μm sections were cut from frozen frontal cortical tissue of an AD patient at Braak stage IV. The same procedure was followed as described above except that the incubation volume was 0.25 ml. The levels of tau species, Aβ40 and Aβ42 in the media were measured by ELISA as described above.

### Tau degradation by microglia-conditioned media

To prepare microglial-conditioned media (MCM), microglia were isolated as described above and incubated in the growth media for 24 hrs. The MCM was harvested and cleared by centrifugation at 1500 rpm. Brain sections from a 6-7 month old P301S transgenic mouse prepared as described above were then incubated with control media (prepared in parallel to MCM but without the addition of cells) or MCM for 24 hrs. Tau levels in the media were assessed by ELISA as described.

### FITC conjugation of AT8 antibody

AT8 was conjugated with FITC using a conjugation kit from Abcam (cat No:102884) following the protocol provided by the vendor. Briefly, 10 μg of AT8 antibody (1 μg/μl) was first gently mixed with 1 μl modifier provided and then added directly into the lyophilized FITC labeling reagent followed by incubation in the dark at 4 °C overnight. The AT8-FITC conjugate was ready for use after adding 1 μl quencher reagent to quench the un-conjugated free FITC.

### Tissue labeling with FITC-conjugated AT8

The brain stem and spinal cord were dissected from 6-7 month old P301S homozygous transgenic mice and age-matched wild-type C57BL/6 mice. The brain tissue was sectioned into 1 mm sections using a McIlwain Tissue Chopper and then incubated with FITC-conjugated AT8 antibody (50 μg/ml in PBS) in the presence of 1% Triton x-100 for 1 hr at 37 °C. The sections were then extensively washed three times in PBS to remove unbound FITC-conjugated AT8.

### FITC-AT8-Tau complex internalization by live microglia

Primary microglia from C57BL/6 mice were isolated as described above and plated onto 8-well Lab-Tek chamber slides (Nunc) in culture medium at a density of 1.5 × 10^5^ cells/well. After the cells attached to the slide (2 hrs post-seeding), the medium was replaced with OPTI-MEM medium (HEPES buffer) containing 0.2% BSA. Then the 1 mm FITC-AT8-labeled tissue section was added to the well followed by incubation at 37 °C for 1 hr. The fluorescent image was collected at 488-nm excitation/538-nm emission using a Leica TCS SP5 spectral confocal microscope under 63 × objective at low light/laser intensities to prevent photo-toxicity.

### Labeling of microglia by CD11b-FITC

Primary microglia were seeded on 8-well Lab-Tek chamber slides (Nunc) at 1.5 × 10^5^/well overnight and then fixed with 4% paraformaldehyde before incubation with CD11b-FITC antibody for 15 min at 4 °C (Sigma, #L2895) in culture medium. Images were collected using DS-Qi1MC of Nikon Eclipse-80i. The percentage of CD11b-FITC-labeled cells was quantified by manual counting for 1000 cells from images taken from random fields and the microglial cultures were ≥98.7% pure.

### Immunohistochemistry

To detect tau uptake by microglia, microglia were fixed with 4% paraformaldehyde (PFA)/PBS for 15 min at room temperature after incubation with 1μg/ml SI-tau for the indicated times, and subjected to indirect immunohistochemistry staining using the 1 ug/ml tau antibodies: AT8 (Thermo Scientific #MN1020); MC1 (provided by Dr. Peter Davies), AT180 (Thermo Scientific #MN1040), AT270 (Thermo Scientific #1050). We followed similar procedures provided by the MOM kit protocol (Vector Labs) with minor modification. Briefly, PFA-fixed microglia were first blocked with mouse IgG and Superblock solution for 30 min (Pierce#37535) and then incubated with AT8 antibody (Thermo Scientific Pierce, 1:500) for 2 hrs at room temperature. After washing extensively (5×) with TBS/0.5% Triton x-100 for 5 min, microglia were incubated with Alexa Fluor 488-conjugated AffiniPure donkey anti-mouse IgG (H + L) (Jackson immunoresearch #715-545-150, 1:200) at room temperature for 30 min. After washing with PBS, cells were stained with red-fluorescence Alexa Fluor594 wheat germ agglutinin (WGA) for plasma membrane and Hoechst 33342 dye for nucleus (Molecular Probes, Image-iT LIVE plasma membrane and nuclear labeling kit #I34406). The fluorescent images were captured using a Leica TCS SP5 spectral confocal microscope with a 63x objective.

To quantify NFTs in P301S transgenic mouse brain sections after incubation with or without microglia, brain sections were fixed with 4% PFA/PBS overnight at 4 °C and stained with AT8 antibody (1:500) using the MOM kit (Vector Laboratories, #PK2200). The color image of each brain section was captured (eight brain sections per treatment group) with a DS-FiL camera and an Eclipse-80i imaging system (Nikon). The number of NFTs from each brain section in the two treatment groups was quantified using NIS Elements AR4.10.01 software after the black/white images were captured with DS-Qi1MC of Nikon Eclipse-80i.

### Statistical analyses

To compare differences between the experimental groups, a two-tailed *t* test or one-way ANOVA was performed using GraphPad Prism software.

## Additional Information

**How to cite this article**: Luo, W. *et al.* Microglial internalization and degradation of pathological tau is enhanced by an anti-tau monoclonal antibody. *Sci. Rep.* **5**, 11161; doi: 10.1038/srep11161 (2015).

## Supplementary Material

Supplementary Information
