# Supplementary material for: Microglial internalization and degradation of pathological tau is enhanced by an anti-tau monoclonal antibody
Source: Sci Rep. 2015 Jun 9;5:11161. doi: 10.1038/srep11161 (PMC4460904; doi:10.1038/srep11161)

**Title: “Microglial internalization and degradation of pathological tau is enhanced by an anti-tau monoclonal antibody”**

Wenjie Luo**^*^**, Wencheng Liu, Xiaoyan Hu, Mary Hanna, April Caravaca, Steven M. Paul**^*^**

Appel Alzheimer’s Disease Research Institute, Brain and Mind Research Institute, Weill Cornell Medical College of Cornell University, 413 East 69^th^ Street, New York, NY10021

**^*^**Correspondence should be addressed to W.L. (wel2009@med.cornell.edu) or S.M.P. (email: [smpaulmd@gmail.com](mailto:smpaulmd@gmail.com))

**Supplemental Information**

**Supplementary Figure Legends:**

**Figure S1:** Internalization of SI-tau by microglia. Primary murine microglia were incubated with 1 μg/ml SI-tau for 120 min and imaged using confocal microscopy after immunostained with AT180 or AT270 antibody. Plasma membrane was stained by red-fluorescent Alexa Fluor594 wheat germ agglutinin (WGA) and nuclei were stained by blue Hoechst 33342 dye. Note the intracellular localization of AT180 or AT270-positive tau punctate (arrows) in microglia. Large tau aggregates attached to microglial surface were also observed (arrowheads). Scale bar represents 20μm.

**Figure S2:** The levels of total tau and AT8-positive tau in the medium incubated with unfixed frozen P301S transgenic brain sections. (**a**) The amount of total human tau and AT8-positive tau released from P301S brain section remains stable in the medium from 1 h to 48 hrs incubation times. (**b**) The quantification of tau levels in the medium after incubation for 1 h with P301S brain section in the presence or absence of microglia. No significant difference was observed between medium alone group and microglia group.

**New Supplementary Figure S1**

**
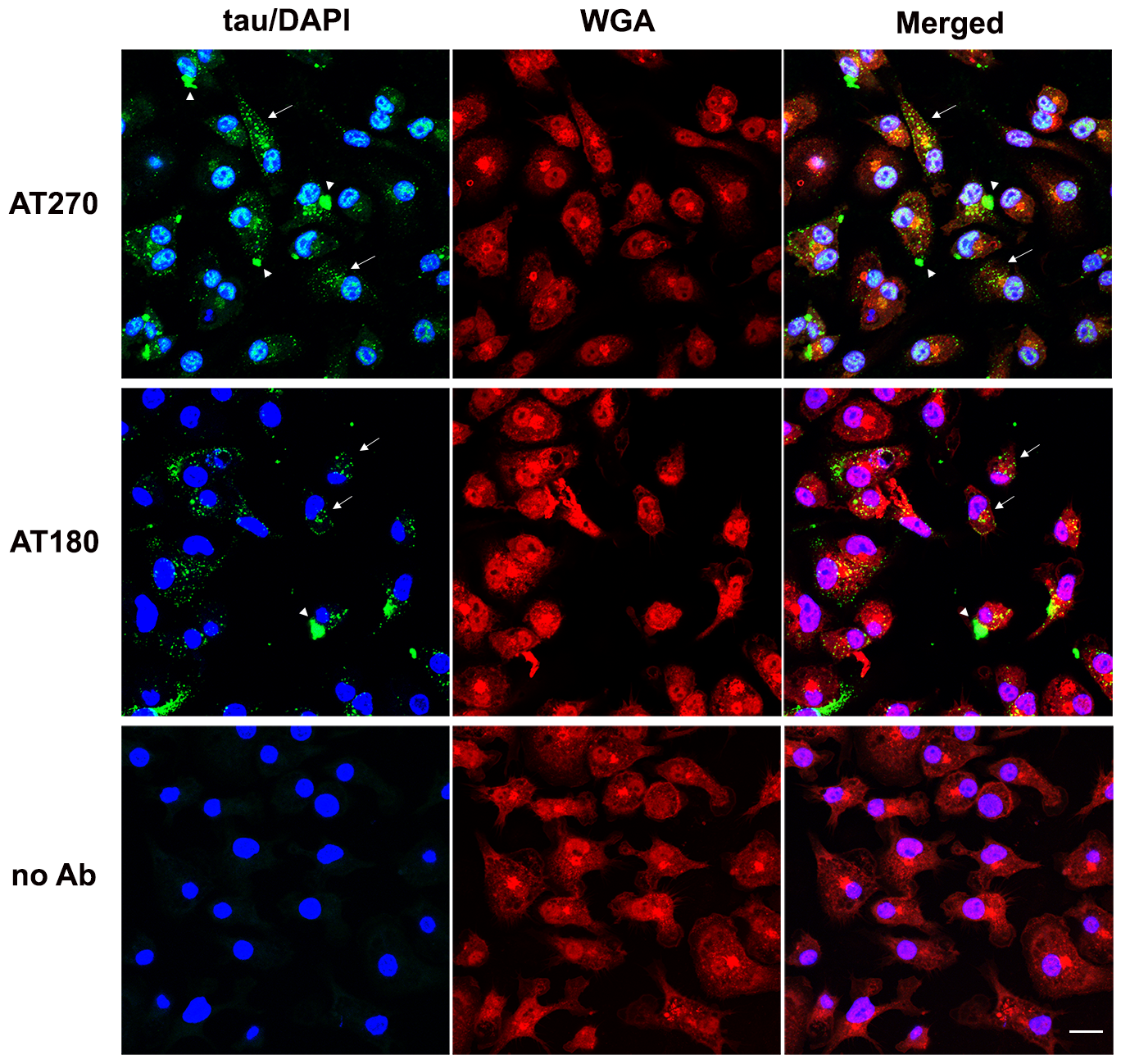
**

**New Supplementary Figure S2**


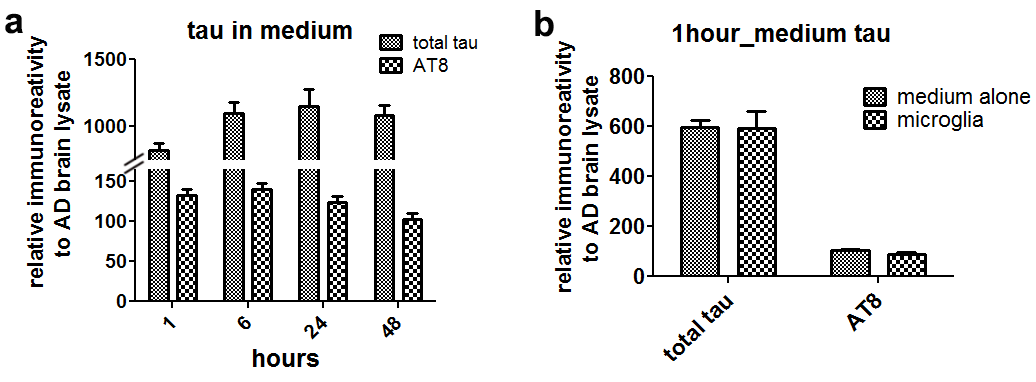

Supplement: Supplementary Information [file srep11161-s1.doc]
